# Prospects of Improving Nitrogen Use Efficiency in Potato: Lessons From Transgenics to Genome Editing Strategies in Plants

**DOI:** 10.3389/fpls.2020.597481

**Published:** 2020-12-23

**Authors:** Jagesh Kumar Tiwari, Tanuja Buckseth, Rajesh Kumar Singh, Manoj Kumar, Surya Kant

**Affiliations:** ^1^Indian Council of Agricultural Research (ICAR)-Central Potato Research Institute, Shimla, India; ^2^Agriculture Victoria, Grains Innovation Park, Horsham, VIC, Australia; ^3^Faculty of Veterinary and Agricultural Sciences, Centre for Agricultural Innovation, School of Agriculture and Food, The University of Melbourne, Melbourne, VIC, Australia

**Keywords:** CRISPR-Cas9, nitrogen use efficiency, plant, potato, transgenic

## Introduction

Feeding the world population increasing from current 7.7 to 9.7 billion by 2050 is a big challenge (United Nations, 2019). This is further serious in developing countries where degradation of soil health, increasing fertilizers cost and reducing cultivable lands are the major constraints (St Clair and Lynch, 2010). Presently, 119.41 million tons of nitrogen (N) fertilizers are applied worldwide in crops to achieve desirable yield (FAO, 2018). Plant N uptake, transport, utilization/assimilation and remobilization are controlled by a complex network of genes involved in these biological processes. Significant research advancements have been made in nitrogen use efficiency (NUE) in plants like *Arabidopsis*, rice, maize and wheat (Li et al., 2017), and physiological and molecular mechanisms underlying N pathways have been elucidated in plants (Kant et al., 2011). Although, many studies have been undertaken in different N regimes and candidate genes have been identified for increasing NUE but success in achieving N-use efficient genotypes is limited due to its complex genetics and genotype by environment interaction (Baligar et al., 2001). Interestingly, a considerable number of transgenic plants with increased NUE have been developed in cereals (Li et al., 2020). Notably, progress in CRISPR/Cas9 [clustered regularly interspaced short palindromic repeat (CRISPR)/CRISPR-associated nuclease 9] genome editing combined with base-editing technology provides a great opportunity for enhancing NUE in plants (Khatodia et al., 2016; Li et al., 2018).

Potato (*Solanum tuberosum* L.) is the fourth most important food crop of the world after rice, wheat and maize. Potato is an N fertilizer intensive crop that requires 180–240 kg N/ha fertilizers to produce high tuber yield (35–45 t/ha); of total applied N, plants acquire only 40–50% and remaining N is lost in environment (Trehan and Singh, 2013). Owing to the adverse impacts and high production cost caused by excess N fertilizers application, improving NUE of plant is an environmental-friendly approach to achieve sustainable crop yield (Fageria et al., 2008). This opinion article highlights prospects for improving NUE in potato based on the lessons learnt from the transgenics to the CRISPR/Cas9 genome editing research in plants.

## Applications of Transgenics and CRISPR-Cas Technologies for Improving NUE in Plants

Transgenic technology has been applied in plants to create genetically modified organism (GMO) by overexpression or knockout/silencing of genes. Genes have been transferred within or across the species to introduce new or enhance/alter endogenous gene expression (Good et al., 2007). Whereas, gene silencing (RNAi) process inhibits gene expression or translation by disrupting targeted mRNA (Liang et al., 2014). N metabolism pathways genes such as nitrate or ammonium transporters, assimilation genes and transcription factors (TFs) have been manipulated for improving NUE in cereals (Li et al., 2020). Generally, overexpression of genes driven by the constitutive (Ubiquitin and CaMV35S) or tissue-specific (e.g., *OsNAR2.1*) promoters has been deployed to develop N-use efficient transgenic plants (Chen J. et al., 2016). Hu et al. (2015) have demonstrated overexpression of *OsNRT1.1B* allele of *indica* rice into *japonica* to increase NUE. RNAi technology has been deployed to knockdown the NAC-like TF *OsNAP* to improve NUE in rice (Liang et al., 2014).

The recently discovered CRISPR/Cas9 system has revolutionized the plant research. CRISPR/Cas9 (type-II, originates from *Streptococcus pyogenes*) is an adaptive immunity found in bacteria or archaea to combat with invading nucleic acids (Khatodia et al., 2016). The unprecedented advances in CRISPR/Cas9 facilitate an easy, versatile and robust technology to accelerate genomics-assisted crop improvement. CRISPR/Cas9 has been successfully deployed to edit N transporter gene to enhance NUE by introgression of *NRT1.1B*-*indica* allele into *japonica* rice (Li et al., 2018). Until now, CRISPR/Cas9 has been mostly applied to mutate negative regulators, instead of overexpression of positive regulators. The gene *BT2*, a member of the Bric-a-Brac/Tramtrack/Broad gene family, suppresses nitrate uptake and NUE; and overexpression of BT2 decreased NUE in rice under low nitrate by decreasing expression of *NRT2.1* and *NRT2.4* genes (Araus et al., 2016). Further, symbiotic N fixation (SNF)-associated genes have also been inactivated by CRISPR/Cas9 in *Lotus japonicus* (Wang et al., 2016), and thus progress in genome editing would accelerate SNF research in legumes and non-legumes. Recently, cytosine- and adenine- base editors (CBEs/ABEs) called base-editing, based on CRISPR/Cas9, have emerged as a newer technology for precise modification of nucleotides [C to T (or G to A), and A to G (or T to C)] for gain or loss of gene functions in eukaryotes (Li et al., 2018; Mishra et al., 2020). The base-editing has been demonstrated in rice for nitrate transporter gene *OsNRT1.1B* to improve NUE (Lu and Zhu, 2017; Zong et al., 2018). Collectively, the successful examples of a few N-use efficient plants (transgenics and genome-edited) are summarized in Supplementary Table 1.

## Recent Results in Crops NUE Modification

### Increasing N Uptake and Transport Efficiency

A number of N transporter genes such as low-affinity nitrate transporter *NRT1.1b* (Fan et al., 2015; Hu et al., 2015), high-affinity nitrate transporters *NRT2.1* (Chen J. et al., 2016; Chen et al., 2017), *NAR2.1* and *NRT2.3a* (Chen et al., 2020), *NRT2.3* (Fu et al., 2015), and *NRT2.3b* (Fan et al., 2016), peptide transporter *PTR9* (Fang et al., 2013), ammonium transporter *AMT1;1* (Ranathunge et al., 2014), and quantitative trait loci *qNGR9*, synonymous with gene *DEP1* (Sun et al., 2014) have shown to enhance NUE in rice. Similarly, TFs such as *MADS25* (Yu et al., 2015) and *NAC2-5A* (He et al., 2015) have also been found effective in developing N use efficient rice and wheat, respectively. The roles of microRNA miR166 targeting Dof TF *RDD1* have also been confirmed to promote ammonium uptake and transport in rice (Iwamoto and Tagiri, 2016). Recently, functions of several genes have been elucidated in plants for high NUE such as nitrate transporter *OsNPF4.5* (Wang et al., 2020), *NAC42*-activated nitrate transporter (Tang et al., 2019) and nitrate reductase gene *OsNR2* (Gao et al., 2019). Collectively, genetic engineering in N transporters have been proven successful to increase plant growth, root architecture, N uptake and transport and total N content, and thus improved NUE of plants (Supplementary Table 1).

### Increasing Plant N Utilization and Remobilization Efficiency

Several genes have been engineered to enhance N utilization efficiency in plants. For example NIN-LIKE PROTEIN 7 (*NLP7*) (Yu et al., 2016), asparagine synthetase *ASN1* (Lam et al., 2003), autophagy-related gene *ATG7-1* (Wada et al., 2015) and glutamine synthetase *GS1;2* (Brauer et al., 2011) have improved NUE in Arabidopsis/rice. The functions of TFs such as bZIP *AtTGA4* (Zhong et al., 2015), *HY5* (Chen X. B. et al., 2016), NAC-like *NAP* (Liang et al., 2014), Dof1 *ZmDof1* (Yanagisawa et al., 2004; Kurai et al., 2011), NAC1-type *NAC-S* (Zhao et al., 2015) and Nuclear Factor Y *NFYA-B1* (Qu et al., 2015) have been demonstrated in development of N-use efficient plants of Arabidopsis/rice/wheat. Importantly, barley *AlaAT* (*alanine aminotransferase*) has most successful in increasing NUE in rice (Shrawat et al., 2008), canola (Good et al., 2007) and sugarcane (Snyman et al., 2015). The miR166 targeting Dof TF *RDD1* enhances transport of nutrients including ammonium and sucrose, N uptake and content, and grain yield under low N in rice (Iwamoto and Tagiri, 2016) (Supplementary Table 1).

## Results and Targets for Improving NUE in Potato

In potato, several studies have reported on application of soil and agronomic practices for N management (review by Trehan and Singh, 2013), but very limited on genomics uses to enhance NUE. Hence, knowledge about genes and regulatory elements such as TFs and microRNAs (miRNAs) are important to improve NUE. Moreover, the underlying molecular and physiological mechanisms and genetic factors remain elusive in potato for root system architecture, carbon-nitrogen economy and N metabolism (uptake, transport, utilization and remobilization). Recently, we have reviewed application of integrated genomics, physiology and breeding approaches for improving NUE (Tiwari et al., 2018) and traits phenotyping under aeroponic in potato (Tiwari et al., 2020d). Further, recent studies provide information about the genes and miRNAs associated with N stress in potato (Tiwari et al., 2020a,b,c; Zhang et al., 2020). Potato is highly amenable to tissue culture and therefore transgenics protocols are well established. Also, CRISPR/Cas9 tool has been applied in potato such as creation of homozygous mutants, knockdown of steroidal glycoalkaloids, caroteinoid biosynthesis and phosphate transport (review by Nadakuduti et al., 2018; Dangol et al., 2019).

**Figure 1:**
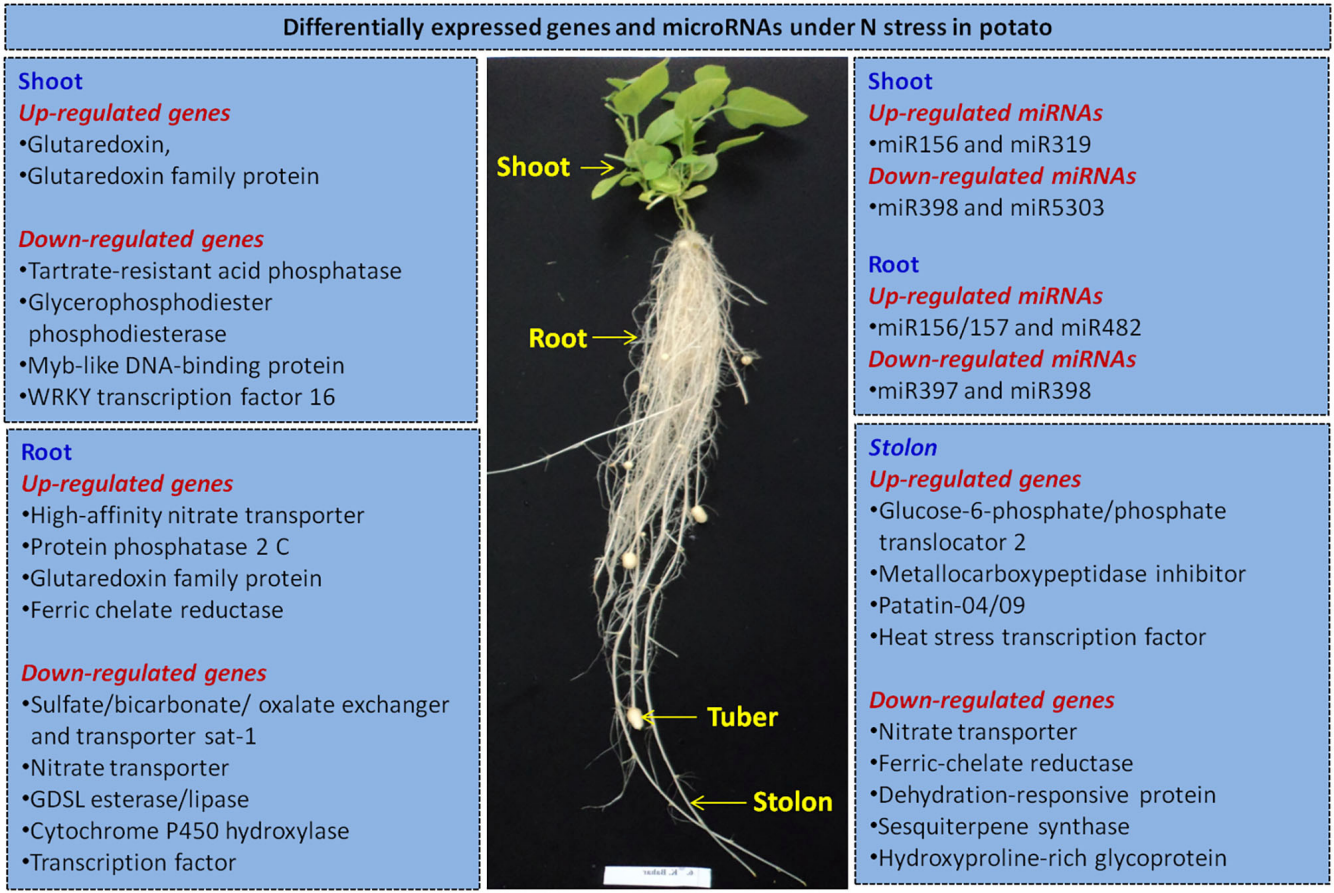
An outline of selected differentially expressed genes identified in potato under N stress based on transcriptome (RNA-seq and small RNA) sequencing of plant grown in aeroponic culture (Tiwari et al., 2020a,b,c). It summarizes the potential candidate genes, transcription factors and microRNAs in different potato tissues (root, shoot/leaf, and stolon) for improving nitrogen use efficiency in potato by gene manipulation via transgenics and/or CRISPR/Cas9 genome editing coupled with based-editing technologies.

We summarize here potential candidate genes for improving NUE in potato based on the recent research (Tiwari et al., 2020a,b,c; Zhang et al., 2020). Our studies indicate that in potato roots, high-affinity nitrate transporters are the key candidate genes for manipulation in N uptake and transport. Moreover, genes like ferric chelate reductase, protein phosphatase 2 C, glutaredoxin, GDSL esterase/lipase, cytochrome P450 hydroxylase and TFs also appear important in roots. In stolons, nitrate transporter, urea active transporter and sodium/proline symporter facilitate N transport. We have also elucidated miRNAs (up-regulated: miR156/157 and miR482, and down-regulated: miR397 and miR398) in roots under N stress. Further, glutaredoxin gene family has been found the most prominent candidate gene under N stress in shoots. Another study shows effect of overexpression of glutaredoxin gene *OsGRX6* on signaling and N status in rice (El-Kereamy et al., 2015). We have identified tartrate-resistant acid phosphatase, glycerophosphodiester phosphodiesterase and TFs (Myb and WRKY), and miRNAs (up-regulated: miR156 and miR319, and down-regulated: miR398 and miR5303) in shoots under N stress. Indeed, stolon formation is a critical stage of tuber formation in potato. Hence, carbohydrate metabolism genes like glucose-6-phosphate/phosphate translocator 2 and glucose-1-phosphate adenylyltransferase, and amino acid synthesis genes such as 2-oxoglutaratedependent dioxygenase, malate synthase and branched-chainamino-acid aminotransferase play crucial roles in potato tuberization. Likewise, inhibitors (cysteine protease and metallocarboxypeptidase), storage protein (patatin), TFs (heat stress, BTB/POZ and LOB domains, F-box), dehydration-responsive protein RD22 and hydroxyproline-rich glycoprotein are essentially involved in potato tuberization. Recently, Zhang et al. (2020) have observed key roles of nitrate transporters (*StNRT2*. *4, StNRT2*. *5*, and *StNRT2*. *7)*, glutamate dehydrogenase, glutamine synthetase and carbonic anhydrase in N metabolism in potato. Thus, like other plants, gene manipulation of N transporters in roots and assimilatory genes of carbohydrate and amino acids metabolism in shoots/stolons, and TFs (Myb and WRKY) could be manipulated by constitutive or tissue-specific promoters. Further, gene knockdown could be applied via RNAi (miR156, miR397, miR398, miR319, and miR482) or others targeting N pathways genes for improving NUE in potato (Figure 1). Moreover, CRISPR/Cas9 has been deployed in potato for multiple genes like *Acetolactate synthase 1* (Butler et al., 2015) and *granule*-*bound starch synthase* (*GBSS*) (Andersson et al., 2017). Overall, candidate genes, TFs and miRNAs could be attempted for genetic manipulation to increase NUE in potato via transgenic or CRISPR/Cas9 or base-editing technologies.

Potato is a tetraploid and therefore application of CRISPR/Cas9 is more challenging. Albeit, all four alleles of *StGBSS* gene in potato have been knocked out and genome-edited mutants have been regenerated (Andersson et al., 2017). Moreover, various transformation methods like *Agrobacterium*, geminivirus replicon, protoplasts and polyethylene glycol have been suggested for Cas9 application in potato (Butler et al., 2015; Nadakuduti et al., 2018), of which protoplasts is an excellent one (Andersson et al., 2017). Further, suitable sgRNA promoters like *Arabidopsis* (*AtUp*) and potato (*StU6p*), and plant promoters like *CAMV35S* have been suggested for potato (Belhaj et al., 2013). Nevertheless, selection of target gene, design of guide RNA, efficient CRISPR/Cas9, plant transformation and off-target mutants are the major issues of genome editing in potato.

## Conclusions

Plant N metabolism involves a network of genes associated in N uptake, transport, utilization, remobilization and storage processes. NUE is a complex multigenic trait and therefore its improvement becomes difficult particularly in tetraploid potato. However, a substantial success has been achieved through transgenic and little via CRISPR/Cas in plants. CRISPR/Cas9 has been mostly applied to negative regulators of genes, and therefore in future it is expected to discover such additional genes. Here, we have suggested a few candidate genes based on our research findings for improving NUE in potato applying transgenics or CRISPR/Cas9 technologies. Further, strengthening the knowledge on genes, TFs, and microRNAs and elucidating underlying molecular and physiological mechanisms of N pathways are vital for NUE research. Collectively, CRISPR/Cas9 coupled with base-editing strategies represents an invaluable system for precise genome editing. Nonetheless, robust Cas9 array system with multiplexing of targets, transformation and regeneration, phenotypes and people awareness would be challenges in genome editing research.

## Author Contributions

JT conceived the idea and wrote the manuscript. JT, TB, RS, and MK performed research work and improved the manuscript. SK critically read and edited the manuscript. All authors contributed to the article and approved the submitted version.

## Conflict of Interest

The authors declare that the research was conducted in the absence of any commercial or financial relationships that could be construed as a potential conflict of interest.

## References

[B1] Andersson M. Turesson H. Nicolia A. Fält A. S. Samuelsson M. Hofvander P. (2017). Efficient targeted multiallelic mutagenesis in tetraploid potato (*Solanum tuberosum*) by transient CRISPR-Cas9 expression in protoplasts. Plant Cell. Rep. 36, 117–128. 10.1007/s00299-016-2062-3 27699473 PMC5206254

[B2] Araus V. Vidal E. A. Puelma T. Alamos S. Mieulet D. Guiderdoni E.. (2016). Members of BTB gene family of scaffold proteins suppress nitrate uptake and nitrogen use efficiency. Plant Physiol. 171, 1523–1532. 10.1104/pp.15.01731 27208309 PMC4902579

[B3] Baligar V. C. Fageria N. K. He Z. L. (2001). Nutrient use efficiency in plants. Commun. Soil Sci. Plant Anal. 32, 921–950. 10.1081/CSS-100104098

[B4] Belhaj K. Chaparro-Garcia A. Kamoun S. Nekrasov V. (2013). Plant genome editing made easy: targeted mutagenesis in model and crop plants using the CRISPR/Cas system. Plant Methods 9, 39–47. 10.1186/1746-4811-9-39 24112467 PMC3852272

[B5] Brauer E. K. Rochon A. Bi Y. M. Bozzo G. G. Rothstein S. J. Shelp B. J. (2011). Reappraisal of nitrogen use efficiency in rice overexpressing glutamine synthetase1. Physiol. Plant 141, 361–372. 10.1111/j.1399-3054.2011.01443.x 21214879

[B6] Butler N. M. Atkins P. A. Voytas D. F. Douches D. S. (2015). Generation and inheritance of targeted mutations in potato (*Solanum tuberosum* L.) using the CRISPR/Cas system. PLoS ONE 10:e0144591. 10.1371/journal.pone.0144591 26657719 PMC4684367

[B7] Chen J. Fan X. Qian K. Zhang Y. Song M. Liu Y.. (2017). *pOsNAR2.1:OsNAR2.1* expression enhances nitrogen uptake efficiency and grain yield in transgenic rice plants. Plant Biotechnol. J. 15, 1273–1283. 10.1111/pbi.12714 28226420 PMC5595721

[B8] Chen J. Liu X. Liu S. Fan X. Zhao L. Song M.. (2020). Co-overexpression of *OsNAR2.1* and *OsNRT2.3a* increased agronomic nitrogen use efficiency in transgenic rice plants. Front. Plant Sci. 11:1245. 10.3389/fpls.2020.01245 32903417 PMC7434940

[B9] Chen J. Zhang Y. Tan Y. Zhang M. Zhu L. Xu G.. (2016). Agronomic nitrogen-use efficiency of rice can be increased by driving *OsNRT2.1* expression with the OsNAR2.1 promoter. Plant Biotechnol. J. 14, 1705–1715. 10.1111/pbi.12531 26826052 PMC5066696

[B10] Chen X. B. Yao Q. F. Gao X. H. Jiang C. F. Harberd N. P. Fu X. D. (2016). Shoot-to-root mobile transcription factor *HY5* coordinates plant carbon and nitrogen acquisition. Curr. Biol. 26, 640–646. 10.1016/j.cub.2015.12.066 26877080

[B11] Dangol S. D. Barakate A. Stephens J. Çaliskan M. E. Bakhsh A. (2019). Genome editing of potato using CRISPR technologies: current development and future prospective. Plant Cell Tissue Organ. Cult. 139, 403–416. 10.1007/s11240-019-01662-y

[B12] El-Kereamy A. Bi Y. M. Mahmood K. Ranathunge K. Yaish M. W. Nambara E.. (2015). Overexpression of the CC-type glutaredoxin, *OsGRX6* affects hormone and nitrogen status in rice plants. Front. Plant Sci. 6:934. 10.3389/fpls.2015.00934 26579177 PMC4630655

[B13] Fageria N. K. Baligar V. C. Li Y. C. (2008). The Role of nutrient efficient plants in improving crop yields in the twenty first century. J. Plant Nutr. 31, 1121–1157. 10.1080/01904160802116068

[B14] Fan X. Feng H. Tan Y. Xu Y. Miao Q. Xu G. (2015). A putative 6-transmembrane nitrate transporter *OsNRT1.1b* plays a key role in rice under low nitrogen. J. Integr. Plant Biol. 58, 590–599. 10.1111/jipb.12382 26220694 PMC5054920

[B15] Fan X. Tang Z. Tan Y. Zhang Y. Luo B. Yang M. (2016). Overexpression of a pH-sensitive nitrate transporter in rice increases crop yields. Proc. Natl. Acad. Sci. U. S. A. 113, 7118–7123. 10.1073/pnas.1525184113 27274069 PMC4932942

[B16] Fang Z. Xia K. Yang X. Grotemeyer M. S. Meier S. Rentsch D.. (2013). Altered expression of the *PTR/NRT1* homologue *OsPTR9* affects nitrogen utilization efficiency, growth and grain yield in rice. Plant Biotechnol. J. 11, 446–458. 10.1111/pbi.12031 23231455

[B17] FAO (2018). World Fertilizer Trends and Outlook to 2018. Rome: Food and Agriculture Organization of the United Nations.

[B18] Fu Y. Yi H. Bao J. Gong J. (2015). *LeNRT2.3* functions in nitrate acquisition and long-distance transport in tomato. FEBS Lett. 589, 1072–1079. 10.1016/j.febslet.2015.03.016 25819437

[B19] Gao Z. Wang Y. Chen G. Zhang A. Yang S. Shang L.. (2019). The *indica* nitrate reductase gene *OsNR2* allele enhances rice yield potential and nitrogen use efficiency. Nat. Commun. 10:5207. 10.1038/s41467-019-13110-8 31729387 PMC6858341

[B20] Good A. G. Johnson S. J. De Pauw M. Carroll R. T. Savidov N. (2007). Engineering nitrogen use efficiency with *alanine aminotransferase*. Can. J. Bot. 85, 252–262. 10.1139/B07-019

[B21] He X. Qu B. Li W. Zhao X. Teng W. Ma W.. (2015). The nitrate inducible NAC transcription factor *TaNAC2-5A* controls nitrate response and increases wheat yield. Plant Physiol. 169, 1991–2005. 10.1104/pp.15.00568 26371233 PMC4634051

[B22] Hu B. Wang W. Ou S. Tang J. Li H. Che R.. (2015). Variation in *NRT1.1B* contributes to nitrate-use divergence between rice subspecies. Nat. Genet. 47, 834–838 10.1038/ng.3337 26053497

[B23] Iwamoto M. Tagiri A. (2016). MicroRNA-targeted transcription factor gene *RDD1* promotes nutrient ion uptake and accumulation in rice. Plant J. 85, 466–477 10.1111/tpj.13117 26729506

[B24] Kant S. Bi Y. M. Rothstein S. J. (2011). Understanding plant response to nitrogen limitation for the improvement of crop nitrogen use efficiency. J. Exp. Bot. 62, 1499–1509. 10.1093/jxb/erq297 20926552

[B25] Khatodia S. Bhatotia K. Passricha N. Khurana S. M. Tuteja N. (2016). The CRISPR/Cas genome-editing tool: application in improvement of crops. Front. Plant Sci. 7:506. 10.3389/fpls.2016.00506 27148329 PMC4835450

[B26] Kurai T. Wakayama M. Abiko T. Yanagisawa S. Aoki N. Ohsugi R. (2011). Introduction of the *ZmDof1* gene into rice enhances carbon and nitrogen assimilation under low-nitrogen conditions. Plant Biotechnol. J. 9, 826–837 10.1111/j.1467-7652.2011.00592.x 21624033

[B27] Lam H. M. Wong P. Chan H. K. Yam K. M. Chen L. Chow C. M.. (2003). Overexpression of the *ASN1* gene enhances nitrogen status in seeds of Arabidopsis. Plant Physiol. 132, 926–935. 10.1104/pp.103.020123 12805621 PMC167031

[B28] Li H. Hu B. Chu C. (2017). Nitrogen use efficiency in crops: lessons from Arabidopsis and rice. J. Exp. Bot. 68, 2477–2488. 10.1093/jxb/erx101 28419301

[B29] Li M. Xu J. Gao Z. Tian H. Gao Y. Kariman K. (2020). Genetically modified crops are superior in their nitrogen use efficiency- a meta-analysis of three major cereals. Sci. Rep. 10:8568. 10.1038/s41598-020-65684-9 32444783 PMC7244766

[B30] Li X. Wang Y. Liu Y. Yang B. Wang X. Wei J.. (2018). Base editing with a Cpf1–cytidine deaminase fusion. Nat. Biotechnol. 36, 324–327. 10.1038/nbt.4102 29553573

[B31] Liang C. Wang Y. Zhu Y. Tang J. Hu B. Liu L.. (2014). *OsNAP* connects abscisic acid and leaf senescence by fine-tuning abscisic acid biosynthesis and directly targeting senescence-associated genes in rice. Proc. Natl. Acad. Sci. U. S. A. 111, 10013–10018. 10.1073/pnas.1321568111 24951508 PMC4103337

[B32] Lu Y. Zhu J. K. (2017). Precise editing of a target base in the rice genome using a modified CRISPR/Cas9 system. Mol. Plant 10, 523–525. 10.1016/j.molp.2016.11.013 27932049

[B33] Mishra R. Joshi R. K. Zhao K. (2020). Base editing in crops: current advances, limitations and future implications. Plant Biotechnol. J. 18, 20–31. 10.1111/pbi.13225 31365173 PMC6920333

[B34] Nadakuduti S. S. Buell C. R. Voytas D. F. Starker C. G. Douches D. S. (2018). Genome editing for crop improvement-applications in clonally propagated polyploids with a focus on potato (*Solanum tuberosum* L.). Front. Plant Sci. 9:1607. 10.3389/fpls.2018.01607 30483283 PMC6243044

[B35] Qu B. He X. Wang J. Zhao Y. Teng W. Shao A.. (2015). A wheat CCAAT boxbinding transcription factor increases the grain yield of wheat with less fertilizer input. Plant Physiol. 167, 411–423. 10.1104/pp.114.246959 25489021 PMC4326744

[B36] Ranathunge K. El-Kereamy A. Gidda S. Bi Y. M. Rothstein S. J. (2014). *AMT1;1* transgenic rice plants with enhanced anitha permeability show superior growth and higher yield under optimal and suboptimal anitha conditions. J. Exp. Bot. 65, 965–979. 10.1093/jxb/ert458 24420570 PMC3935567

[B37] Shrawat A. K. Carroll R. T. DePauw M. Taylor G. J. Good A. G. (2008). Genetic engineering of improved nitrogen use efficiency in rice by the tissue-specific expression of alanine aminotransferase. Plant Biotechnol. J. 6, 722–732. 10.1111/j.1467-7652.2008.00351.x 18510577

[B38] Snyman S. J. Hajari E. Watt M. P. Lu Y. Kridl J. C. (2015). Improved nitrogen use efficiency in transgenic sugarcane: phenotypic assessment in a pot trial under low nitrogen conditions. Plant Cell Rep. 34, 667–669. 10.1007/s00299-015-1768-y 25686580

[B39] St Clair S. B. Lynch J. P. (2010). The opening of Pandora's box: climate change impacts on soil fertility and crop nutrition in developing countries. Plant Soil 335, 101–115. 10.1007/s11104-010-0328-z

[B40] Sun H. Qian Q. Wu K. Luo J. Wang S. Zhang C.. (2014). Heterotrimeric G proteins regulate nitrogen-use efficiency in rice. Nat. Genet. 46, 652–656. 10.1038/ng.2958 24777451

[B41] Tang W. Ye J. Yao X. Zhao P. Xuan W. Tian Y.. (2019). Genome-wide associated study identifies *NAC42*-activated nitrate transporter conferring high nitrogen use efficiency in rice. Nat. Commun. 10:5279. 10.1038/s41467-019-13187-1 31754193 PMC6872725

[B42] Tiwari J. K. Buckseth T. Devi S. Varshney S. Sahu S. Patil V. U.. (2020a). Physiological and genome-wide RNA-sequencing analyses identify candidate genes in a nitrogen-use efficient potato cv. Kufri Gaurav. Plant Physiol. Biochem. 154, 171–183. 10.1016/j.plaphy.2020.05.041 32563041

[B43] Tiwari J. K. Buckseth T. Zinta R. Saraswati A. Singh R. K. Rawat S.. (2020b). Transcriptome analysis of potato shoots, roots and stolons under nitrogen stress. Sci. Rep. 10:1152. 10.1038/s41598-020-58167-4 31980689 PMC6981199

[B44] Tiwari J. K. Buckseth T. Zinta R. Saraswati A. Singh R. K. Rawat S.. (2020c). Genome-wide identification and characterization of microRNAs by small RNA sequencing for low nitrogen stress in potato. PLoS ONE 15:e0233076. 10.1371/journal.pone.0233076 32428011 PMC7237020

[B45] Tiwari J. K. Devi S. Buckesth T. Ali N. Singh R. K. Zinta R. (2020d). Precision phenotyping of contrasting potato (*Solanum tuberosum* L.) varieties in a novel aeroponics system for improving nitrogen use efficiency: in search of key traits and genes. J. Integr. Agric. 19:51–61. 10.1016/S2095-3119(19)62625-0

[B46] Tiwari J. K. Plett D. Garnett T. Chakrabarti S. K. Singh R. K. (2018). Integrated genomics, physiology and breeding approaches for improving nitrogen use efficiency in potato: translating knowledge from other crops. Funct. Plant Biol. 45, 587–605. 10.1071/FP17303 32290962

[B47] Trehan S. P. Singh B. P. (2013). Nutrient efficiency of different crop species and potato varieties-in retrospect and prospect. Potato J. 40, 1–21.

[B48] United Nations (2019). Available online at: https://www.un.org (accessed August 6, 2020).

[B49] Wada S. Hayashida Y. Izumi M. Kurusu T. Hanamata S. Kanno K.. (2015). Autophagy supports biomass production and nitrogen use efficiency at the vegetative stage in rice. Plant Physiol. 168, 60–73. 10.1104/pp.15.00242 25786829 PMC4424030

[B50] Wang L. Wang L. Tan Q. Fan Q. Zhu H. Hong Z.. (2016). Efficient inactivation of symbiotic nitrogen fixation related genes in *Lotus japonicus* using CRISPR-Cas9. Front. Plant Sci. 7:1333. 10.3389/fpls.2016.01333 27630657 PMC5006320

[B51] Wang S. Chen A. Xie K. Yang X. Luo Z. Chen J.. (2020). Functional analysis of the *OsNPF4.5* nitrate transporter reveals a conserved mycorrhizal pathway of nitrogen acquisition in plants. Proc. Natl. Acad. Sci. U. S. A. 117, 16649–16659 10.1073/pnas.2000926117 32586957 PMC7368293

[B52] Yanagisawa S. Akiyama A. Kisaka H. Uchimiya H. Miwa T. (2004). Metabolic engineering with *Dof1* transcription factor in plants: improved nitrogen assimilation and growth under low-nitrogen conditions. Proc. Natl. Acad. Sci. U. S. A. 101, 7833–7838. 10.1073/pnas.0402267101 15136740 PMC419692

[B53] Yu C. Liu Y. Zhang A. Su S. Yan A. Huang L.. (2015). MADS-box transcription factor *OsMADS25* regulates root development through affection of nitrate accumulation in rice. PLoS ONE 10:e0135196. 10.1371/journal.pone.0135196 26258667 PMC4530940

[B54] Yu L. H. Wu J. Tang H. Yuan Y. Wang S. M. Wang Y. P.. (2016). Overexpression of Arabidopsis *NLP7* improves plant growth under both nitrogen-limiting and -sufficient conditions by enhancing nitrogen and carbon assimilation. Sci. Rep. 6:27795. 10.1038/srep27795 27293103 PMC4904239

[B55] Zhang J. Wang Y. Zhao Y. Zhang Y. Zhang J. Ma H.. (2020). Transcriptome analysis reveals Nitrogen deficiency induced alterations in leaf and root of three cultivars of potato (*Solanum tuberosum* L.). PLoS ONE 15:e0240662. 10.1371/journal.pone.0240662 33119630 PMC7595393

[B56] Zhao D. Derkx A. P. Liu D. C. Buchner P. Hawkesford M. J. (2015). Overexpression of a NAC transcription factor delays leaf senescence and increases grain nitrogen concentration in wheat. Plant Biol. 17, 904–913. 10.1111/plb.12296 25545326 PMC4949518

[B57] Zhong L. Chen D. Min D. Li W. Xu Z. Zhou Y.. (2015). *AtTGA4*, a bZIP transcription factor, confers drought resistance by enhancing nitrate transport and assimilation in *Arabidopsis thaliana*. Biochem. Biophys. Res. Commun. 457, 433–439. 10.1016/j.bbrc.2015.01.009 25596127

[B58] Zong Y. Song Q. Li C. Jin S. Zhang D. Wang Y.. (2018). Efficient C-to-T base editing in plants using a fusion of nCas9 and human APOBEC3A. Nat. Biotechnol. 36, 950–953. 10.1038/nbt.4261 30272679

