# Supplementary material for: Prospects of Improving Nitrogen Use Efficiency in Potato: Lessons From Transgenics to Genome Editing Strategies in Plants
Source: Front Plant Sci. 2020 Dec 23;11:597481. doi: 10.3389/fpls.2020.597481 (PMC7785987; doi:10.3389/fpls.2020.597481)
Supplement: Supplementary file 1 [file Data_Sheet_1.docx]

**Supplementary Table S1.** Application of transgenics and CRISPR-Cas9 genome editing technologies for improving nitrogen efficiency in plants.

| Sr. No. | Crop | Gene | Technology | Gene function/Results | Reference |
| --- | --- | --- | --- | --- | --- |
| *N uptake and transport* | | | |  |  |
|  | Rice | *NRT1.1B* | CRISPR/Cas9 | Application of the CRISPR/Cas9 system has been demonstrated in replacement of *NRT1.1B* allele in *japonica* rice by the *indica* allele and enhanced NUE showing possibility of effect of gene alteration in rice. | Li *et al.*, 2018 |
|  | Rice | *NRT1.1B* | Base editing | CRISPR/Cas9 nickase-cytidine deaminase fusion protein in *OsNRT1.1B* gene encoding low-affinity nitrate transporter showed direct conversion of cytosine to thymine (Thr327Met) for improving NUE in rice. | Lu and Zhu, 2017 |
|  | Rice | *NRT1.1B* | Base editing | CRISPR/Cas9 nickase-cytidine deaminase enzyme showed allelic conversion of cytosine to thymine from 3 to 9 positions within the protospacer sequence in rice, wheat and maize. | Zong *et al.*, 2018 |
|  | Rice | *NRT1.1b* | Transgenic | Overexpression of the low-affinity nitrate transporter gene *OsNRT1.1b* increased total N accumulation and plant growth in rice under low N supply with both nitrate and ammonium forms. Whereas, overexpression of *OsNRT1.1a* showed increased N accumulation under high N but not in low N. | Fan *et al.*, 2015 |
|  | Rice | *NRT1.1B* | Transgenic | The near isogenic line of *japonica* rice carrying *OsNRT1.1B-indica* allele showed high nitrate uptake, transport and overexpression of nitrate responsive genes, and increased grain yield from 30.3 to 33.4% under low N conditions in field and thus improved NUE. | Hu *et al.*, 2015 |
|  | Rice | *PTR9* | Transgenic | Overexpression of PTR/NRT (peptide transporter/nitrate transporter 1) gene *OsPTR9* increased ammonium uptake, lateral root growth, number of tillers and panicles and grain yield in transgenic rice and thus enhanced NUE. | Fang *et al.*, 2013 |
|  | Rice | *NRT2.1* | Transgenic | Increased expression of the high-affinity nitrate transporter gene *OsNRT2.1* driven by nitrate inducible promoter *OsNAR2.1* enhanced total biomass, grain yield by 21-38% and higher NUE by 128% in rice. | Chen *et al.*, 2016 |
|  | Rice | *NRT2.3b* | Transgenic | Overexpression of the high-affinity nitrate transporter gene *OsNRT2.3b* increased N uptake, grain yield and NUE by 40% under varied N supplies in field in rice. | Fan *et al.*, 2016 |
|  | Tomato | *NRT2.3* | Transgenic | Overexpression of the low-affinity nitrate transporter *LeNRT2.3* gene in tomato increased nitrate uptake, transport, biomass and fruit weight under hydroponic culture. | Fu *et al.*, 2015 |
|  | Rice | *AMT1;1* | Transgenic | Overexpression of the ammonium transporter gene *OsAMT1;*1 enhanced ammonium uptake and its content in shoots and roots, plant growth, gene expression, N assimilates, starch, sugar and grain yield under suboptimal as well as optimal nitrogen in rice. | Ranathunge *et al.*, 2014 |
|  | Rice | *DEP1* and  *qNGR9* | Transgenic | A major NUE quantitative trait loci *qNGR9,* synonymous with *DEP1 (DENSE AND ERECT PANICLE 1)*, regulates panicle architecture in rice. *DEP1* gene interacting with G protein (γ subunit) increased plant growth, N uptake, N assimilation, harvest index and grain yield at moderate N level in rice. | Sun *et al.,* 2014 |
|  | Rice | *MADS25* | Transgenic | Overexpression of *OsMADS25* transcription factor in rice increased root growth (primary root length, and lateral root length and number), shoot biomass, nitrate uptake by higher gene expression of nitrate transporters, nitrate accumulation under high nitrate supply and improved NUE in rice. | Yu *et al.,* 2015 |
|  | Wheat | *NAC2-5A* | Transgenic | NAC transcription factor *TaNAC2-5A* directly binds to the promoter region of nitrate transporter and glutamine synthetase gene in plant. Overexpression of *TaNAC2-5A* increased root growth (lateral root branching), nitrate uptake and transport, N accumulation shoot and grains, N harvest index and improved NUE under low and high N in field trial. | He *et al.*, 2015 |
| *N utilization/assimilation and remobilization* | | | |  |  |
|  | Arabidopsis | *NLP7* | Transgenic | Overexpression of *AtNLP7* (NIN-LIKE PROTEIN 7) gene increased plant biomass, N uptake, N metabolites, total N content, expression of genes involved in N signaling and assimilation, photosynthetic rate and carbon assimilation under low and high N conditions and enhanced NUE in Arabidopsis. *AtNLP7*also improved plant growth and NUE in tobacco. | Yu *et al.*, 2016 |
|  | Arabidopsis | *ASN1* | Transgenic | Overexpression of the asparagine synthetase *ASN1* gene increased soluble seed protein content, total protein content from acid-hydrolyzed seeds, high N status and asparagine content in seed, and N stress tolerance in seedlings. | Lam *et al.,* 2003 |
|  | Rice | *ATG7-1* | Transgenic | Autophagy-related (ATG) genes are essential for Rubisco degradation and nitrogen remobilization from senescent leaves to new tissues or seeds. An autophagy-disrupted mutant *Osatg7-1* showed reduced plant biomass and NUE, and high N concentration in the senescent leaves. | Wada *et al.*, 2015 |
|  | Rice | *GS1;2* | Transgenic | Overexpression of cytosolic glutamine synthetase gene *GS1;1* enhanced harvest index, N harvest index and N utilization efficiency in rice under sufficient N but no improvement under limited N conditions. | Brauer *et al.*, 2011 |
|  | Arabidopsis | *TGA4* | Transgenic | Overexpression of the bZIP transcription factor *AtTGA4* (TGACG motif binding factor 4) increased nitrogen and proline contents, nitrite reductase enzyme activity, expression of high-affinity nitrate transporters *NRT2.1* and *NRT2.2*, and nitrate reductase *NIA1* and *NIA2* genes, and thus showed tolerance to both nitrogen and drought stresses in *Arabidopsis*. | Zhong *et al.*, 2015 |
|  | Arabidopsis | *HY5* | Transgenic | *Arabidopsis* *ELONGATED HYPOCOTYL5* (*HY5*), a bZIP transcription factor, promotes root growth and nitrate uptake in response to light. *HY5* coordinates light-responsive C-N metabolism and N assimilation by positive regulation of *NIA2* (*Nitrate Reductase 2*) and *NRT2.1* genes but negative regulation of *NRT1.1*. | Chen *et al.*, 2016 |
|  | Rice | *NAP* | Transgenic | Knockdown of *Oriza sataiva* NAC-like, activated by apetala3/pistillata (*OsNAP*) transcriptional factor delayed leaf senescence and increased grain-filling period and thereby resulted in 6.3 to 10.3 % increase in grain yield in rice. On the contrary, overexpression of *OsNAP* increased senescence. | Liang *et al.*, 2014 |
|  | Rice | *ZmDof1* | Transgenic | Overexpression of maize transcription factor *ZmDof1* in rice plant increased gene expression of PEPC (phosphoenol pyruvate carboxylase), net photosynthesis rate, C-N metabolism, N assimilation and plant growth under low N supply. | Yanagisawa *et al.*, 2004;  Kurai *et al.*, 2011 |
|  | Wheat | *NAC-S* | Transgenic | Overexpression of a novel NAC1-type transcription factor *TaNAC-S* delayed leaf senescence and increased grain N and protein concentration at same grain yield. | Zhao *et al.*, 2015 |
|  | Wheat | *NFYA-B1* | Transgenic | Overexpression of a low-nitrogen responsive transcription factor *TaNFYA-B1*, Nuclear Factor Y (NF-Y, subunits A, B and C), increased nitrogen uptake and grain yield under varied N supply in field. In contrast, microRNA 169 is negative regulator of low N, and degrades *TaNFYA-B1*. | Qu *et al.*, 2015 |
|  | Rice | *AlaAT* | Transgenic | Overexpression of barley *AlaAT* (*alanine aminotransferase*) driven by *OsAnt1*, rice tissue-specific promoter, increased plant biomass, N metabolites, total N content and N uptake efficiency in rice. | Shrawat *et al.,* 2008 |
|  | Canola | *AlaAT* | Transgenic | Overexpression of barley *AlaAT* driven by canola root-specific promoter *btg26* increased plant biomass, nitrate influx and seed yield under low N in controlled and field conditions, and saving of 40% N fertilizers to achieve equivalent yield. | Good *et al*., 2007 |
|  | Sugarcane | *AlaAT* | Transgenic | Overexpression of *AlaAT* increased gene expression, total dry matter, shoot:root ratio, plant N content, N uptake and utilization efficiency in transgenic sugarcane lines under low N supply in pot culture. | Snyman *et al*., 2015 |
|  | Rice | *RDD1* | Transgenic | Overexpression of rice Dof transcription factor *OsRDD1*, targeted by microRNA miR166, enhanced transport various nutrients including ammonium and sucrose, N uptake, N accumulation and grain yield under low N. | Iwamoto and Tagiri, 2016 |
